# Urethra-sparing prostate cancer radiotherapy: Current practices and future insights from an international survey

**DOI:** 10.1016/j.ctro.2024.100907

**Published:** 2024-12-30

**Authors:** Jennifer Le Guévelou, Paul Sargos, Piet Ost, Filippo Alongi, Stefano Arcangeli, Alejandro Berlin, Pierre Blanchard, Anna Bruynzeel, Olivier Chapet, Alan Dal Pra, Robert T. Dess, Matthias Guckenberger, Andrew Loblaw, Amar U. Kishan, Barbara Jereczek-Fossa, David Pasquier, Mohamed Shelan, Shankar Siva, Alison C. Tree, Costantinos Zamboglou, Stephane Supiot, Vedang Murthy, Thomas Zilli

**Affiliations:** a Department of Clinical Research, Centre Eugène Marquis, Rennes, France; b Laboratoire du traitement du signal et de l’image, Université De Rennes, Rennes, France; c Department of Radiation Oncology, Institut Bergonié, Bordeaux, France; d Department of Radiotherapy, Charlebourg Center, La Garenne-Colombes, France; e Department of Human Structure and Repair, Ghent University, Ghent, Belgium; f Iridium Network, Radiation Oncology, Wilrijk, Belgium; g Advanced Radiation Oncology Department, IRCCS Sacro Cuore Don Calabria Hospital, Cancer Care Center, Italy; h University of Brescia, Brescia, Italy; i Radiation Oncology Department, Fondazione IRCCS San Gerardo dei Tintori, Monza, Italy; j School of Medicine and Surgery, University of Milan Bicocca, Milan, Italy; k Department of Radiation Oncology, Princess Margaret Cancer Center, University Health Network and University of Toronto, Toronto, Ontario, Canada; l Department of Radiation Oncology, Gustave Roussy, Université Paris-Saclay, Villejuif, France; m Amsterdam UMC Location Vrije Universiteit Amsterdam, Department of Radiation Oncology, Cancer Center Amsterdam, De Boelelaan 1117, Amsterdam, Netherlands; n Department of Radiation Oncology, Hôpital Lyon Sud, Lyon, France; o Université Claude-Bernard Lyon 1, Villeurbanne, France; p Department of Radiation Oncology, Sylvester Comprehensive Cancer Center, University of Miami Health Systems, Miami, FL, USA; q Department of Radiation Oncology, University of Michigan, Ann Arbor, MI, USA; r Department of Radiation Oncology, University Hospital Zürich, University of Zürich, Zürich, Switzerland; s Institute of Healthcare Policy and Management, Department of Radiation Oncology, Ontario Institute of Cancer Research, University of Toronto, Toronto, Ontario, Canada; t Department of Radiation Oncology, University of California, Los Angeles, CA, USA; u Department of Oncology and Hemato-oncology, University of Milan, Milan, Italy; v Department of Radiation Oncology, IEO European Institute of Oncology IRCCS, Milan, Italy; w Department of Radiation Oncology, Centre Oscar Lambret, Lille, France; x CRIStAL UMR CNRS 9189, Lille University, Lille, France; y Department of Radiation Oncology, Inselspital, Bern University Hospital, University of Bern, Switzerland; z Division of Radiation Oncology and Sir Peter MacCallum Department of Oncology, Peter MacCallum Cancer Center, University of Melbourne, Melbourne, Victoria, Australia; aa Department of Radiation Oncology, The Royal Marsden NHS Foundation Trust, Sutton, UK; ab The Institute of Cancer Research, London, UK; ac German Oncology Center, European University of Cyprus, 1 Nikis Avenue, 4108, Agios Athanasios, Cyprus; ad Department of Radiation Oncology, University Hospital Freiburg, Robert-Koch-Straße 3, 79106, Freiburg, Germany; ae Radiation Oncology Department, Institut de Cancérologie de l’Ouest, Nantes Saint-Herblain, France; af CNRS US2B, University of Nantes, Nantes, France; ag Department of Radiation Oncology, Tata Memorial Hospital and Advanced Centre for Treatment Research and Education in Cancer (ACTREC), Homi Bhabha National Institute (HBNI), Mumbai, India; ah Department of Radiation Oncology, Oncology Institute of Southern Switzerland, EOC, Bellinzona, Switzerland; ai Faculty of Biomedical Sciences, Università della Svizzera italiana, Lugano, Switzerland; aj Faculty of Medicine, University of Geneva, Geneva, Switzerland

**Keywords:** Urethra, Prostate cancer, SBRT, Radiotherapy, Toxicity

## Abstract

**Purpose:**

In prostate cancer patients, high radiation doses to the urethra have been associated with an increased risk of severe genitourinary toxicity following dose-escalated radiotherapy. Urethra-sparing techniques have emerged as a promising approach to reduce urinary toxicity. This international survey aims to evaluate current global practices in urethra-sparing and explore future directions for the implementation of this technique in external beam radiotherapy (EBRT) for prostate cancer.

**Methods and materials:**

In April 2024, a survey consisting of 20 questions was distributed to 26 international radiation oncology experts in prostate cancer EBRT, with 23 experts participating. The survey focused on clinical scenarios which might take benefit from urethra-sparing, the definition of the urethra and urinary organs-at-risk, and urethral dose constraints.

**Results:**

Magnetic resonance imaging with T2-weighted sequences is the preferred method for urethra contouring (83 % consensus). Based on the experts opinion, urethra-sparing should be considered for prostate cancer EBRT, regardless of pelvic irradiation, except in cases where the tumor is located within 2 mm of the urethra and/or transitional zone, or in T4 disease. Most experts would not apply specific dose constraints to the urethra for either conventional or moderate hypofractionation regimens. When delivering stereotactic body radiotherapy (SBRT), urethra-sparing with dose hotspot limitation (urethra steering) is recommended by 70 % of the experts, in particular when combined with focal boosting (91 %). Urethra dose-reduction is also the favored approach for salvage prostate reirradiation with SBRT (70 % agreement). Large variations exists regarding urethral dose constraints.

**Conclusions:**

Urethra-sparing is a promising technique for the mitigation of urinary toxicity in prostate cancer patients undergoing EBRT, particularly recommended for ultra-hypofractionation and reirradiation with SBRT. The lack of consensus on specific urethral dose constraints and optimal sparing techniques underscores the need for further research to standardize practices in this field.

## Highlights

• Use of MRI is recommended for urethra delineation.
• Urethra-sparing contraindications: tumors <2mm from urethra and T4 tumors invading urinary structures.
• Urethra-sparing techniques are recommended for prostate SBRT with focal boosting.
• Urethra-sparing: considered for standard dose SBRT or reirradiation SBRT by >70% of experts.

## Introduction

Genito-urinary (GU) toxicity represents one of the major concerns following curative external beam radiotherapy (EBRT) for prostate cancer. While advancements in modern EBRT techniques and the integration of image-guided radiation therapy (IGRT) have reduced radiation-induced side effects compared to 3D conformal techniques [1], attempts to achieve further dose escalation with stereotactic body radiotherapy (SBRT) or focal boosting to improve disease control may be counterbalanced by an increased risk of GU toxicity.

**Fig. 1:**
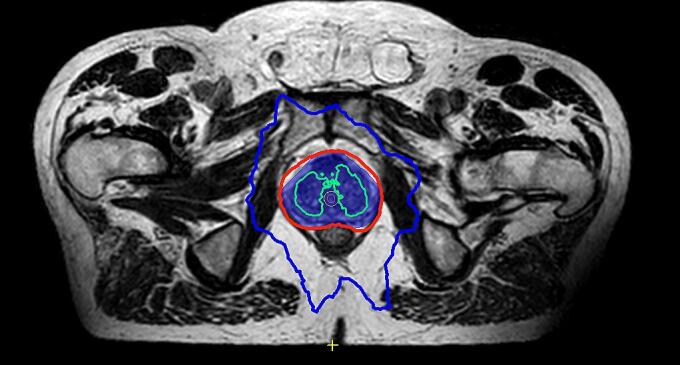
Prostate gland dose distribution by magnetic-resonance guided SBRT with urethra-steering technique (Dose of 36.25 Gy in 5 fractions prescribed to the 80 % isodose line, limiting the hotspots to urethra below 40 Gy). *Legend:* Treatment volumes colors: blue = prostate planning target volume (prostate gland + 2 mm margin); pink = intraprostatic urethra; yellow = urethra PRV (urethra + 2 mm). Isodoses lines: blue = 18 Gy; red = 36.25 Gy; green = 42 Gy.

In addition to adhering to bladder dose constraints, optimizing radiation doses to specific urinary organs at risk (OARs), including the bladder trigone, bladder neck, and urethra, has recently emerged as a promising strategy to further enhance the therapeutic ratio in prostate cancer EBRT [2]. Specifically, high doses to the intraprostatic urethra have been associated have been associated with the occurrence of severe GU toxicity when delivering > 80 Gy with conventional fractionation [3], as well as in patients undergoing SBRT [4]. Urethra-sparing strategies have recently emerged as a potential approach to reduce the risk of severe late GU toxicity of EBRT treatments, either by trying to reduce the maximal doses delivered to the urethra below the dose of prescription to the prostate gland (urethra dose reduction) or by limiting the dose hotspots delivered to the urethra (urethra steering) (Fig. 1) [5]. Despite the emerging literature supporting the role of intraprostatic urethra as an emerging OAR, the current implementation of urethra-sparing techniques in clinical practice remains limited overall, in part because of the large variability that exists in urethra delineation, modalities of sparing, and specific dose constraints.

The purpose of this international survey among prostate cancer experts is to evaluate current urethra-sparing practices worldwide and to assess future insights for implementation of this technique for prostate cancer EBRT.

## Material and methods

Between September and December 2023, two radiation oncologists’ experts on prostate cancer and urethra-sparing techniques (JLG and TZ) designed a survey on use and implementation of urethra-sparing for definitive prostate cancer EBRT. This survey included 20 questions, dealing on the major issues regarding urethra-sparing. The survey was divided in the following sections:

- Clinical situations (9 questions)
- Urethra definition and additional OARs (6 questions)
- Urethra dose-constraints (5 questions)

An external review of the survey was performed by a team of radiation oncologists’ experts in the management of prostate cancer (SS, PS, VM, PO). After validation, the survey was sent by email in April 2024 to a total of 26 international radiation oncologists experts in prostate cancer EBRT using the Google Forms platform. Anonymized responses were collected between May and June 2024. A review of this survey was conducted in June 2024.

## Results

Of the 26 international experts contacted for the survey, 23 participated by completing all the questions. Among these experts, 16 were from Europe, 5 were from North America, and 1 was from Asia and Australia, respectively. Overall, the same response from more than 80 % of the experts was achieved on 2 questions, while a rate between 60 % and 80 % was achieved on 6 questions, as illustrated in Table 1.

**Table 1:** Summarized results of the survey**.**

***Question***	***Proposed answers***	***Answers***	***Preferred answer(s)***
***Clinical situations***			
In your clinical practice, do you treat patients with urethra-sparing?	- Always - Sometimes for selected cases - NeverOther: please specify	- Sometimes for selected cases (56 %) - Always (22 %) - Never (13 %) - Other (9 %)	−
In which clinical situation would you consider a patient for a urethra-sparing technique? (multiple choices accepted)	- Urinary symptoms (score IPSS > 15) - Use of alfa-blockers - Enlarged prostate gland (> 80 cc) - Previous TURP / adenectomy - It depends on the radiotherapy technique and fractionation - I recommend urethra-sparing in all clinical situations - I do not recommend urethra-sparing - Abstain/unqualified to answer - Other: please specify	- It depends on the radiotherapy technique and fractionation (61 %) - Urinary symptoms (score IPSS > 15) (22 %) - I recommend urethra-sparing in all clinical situations (17 %) - Use of α-blockers (13 %) - Enlarged prostate gland (> 80 cc) (13 %) Previous TURP / adenectomy (13 %) - I do not recommend urethra-sparing (4 %) - Other (13 %)	**It depends on the radiotherapy technique and fractionation (61 %)**
In which clinical situation would you exclude a patient from a urethra-sparing technique? (multiple choices accepted)	- Prostate benign hypertrophy - Previous TURP / adenectomy - Tumor located < 2 mm to the urethra and/ or transitional zone - Tumor involving more than 50 % of the prostate gland - T4 tumor (invading urethra, sphincter, bladder) - I recommend urethra-sparing in all clinical situations - I do not recommend urethra-sparing - Abstain/unqualified to answer - Other: please specify	- Tumor located < 2 mm to the urethra and/ or transitional zone (70 %) - T4 tumor (invading urethra, sphincter, bladder) (61 %) - Tumor involving more than 50 % of the prostate gland (22 %) - I do not recommend urethra-sparing (13 %) - I recommend urethra-sparing in all clinical situations (9 %) - Previous TURP / adenectomy (4 %) - Prostate benign hypertrophy (0 %) - Other (4 %)	**Tumor located < 2 mm to the urethra and/ or transitional zone (70 %)** **T4 tumor (invading urethra, sphincter, bladder) (61 %)**
Should urethra-sparing be recommended for all prostate cancer (PCa) NCCN risk-classes?	- Yes, for all NCCN PCa risk classes - No, only for low- and intermediate-risk NCCN PCa - No, only for high-risk NCCN PCa - No, only in selected cases but independently from the risk class - Abstain/unqualified to answer	- Yes, for all NCCN risk classes (35 %) - No, only in selected cases but independently from the risk class (35 %) - No, only for low- and intermediate-risk NCCN PCa (21 %) - No, only for high-risk NCCN PCa (9 %)	−
Target: Would you consider the implementation of urethra-sparing for:	- Prostate-only radiotherapy - Prostate + pelvic radiotherapy - Both situations (prostate with or without pelvis) - I don’t recommend urethra-sparing - Abstain/unqualified to answer	- Both situations (prostate with or without pelvis) (65 %) Prostate-only radiotherapy (26 %) - I don’t recommend urethra-sparing (9 %) - Prostate + pelvic radiotherapy (0 %) - Abstain/unqualified to answer (0 %) - Prostate + pelvic radiotherapy (0 %)	**Both situations (prostate with or without pelvis) (65 %)**
Schedule: For which radiotherapy regimens would you consider the implementation of urethra-sparing? (multiple choices accepted)	- Conventional fractionation / moderate hypofractionated prostate radiotherapy - Conventional fractionation / moderate hypofractionated prostate radiotherapy with dose-escalation on the dominant intraprostatic lesion (DIL) - Prostate SBRT - Prostate SBRT with dose-escalation on the DIL - Salvage prostate re-irradiation with SBRT - I don’t recommend urethra-sparing - Abstain/unqualified to answer	- Prostate SBRT with dose-escalation on the DIL (91 %) - Salvage prostate re-irradiation with SBRT (78 %) - Prostate SBRT (70 %) - Conventional fractionation / moderate hypofractionated prostate radiotherapy with dose-escalation on the dominant intraprostatic lesion (DIL) (48 %) - Conventional fractionation / moderate hypofractionated prostate radiotherapy (9 %) - I don’t recommend urethra-sparing (0 %) - Abstain/unqualified to answer (0 %)	**Prostate SBRT with dose-escalation on the DIL (91 %)** **Salvage prostate re-irradiation with SBRT (78 %)** **Prostate SBRT (70 %)**
Modality: Which urethra-sparing technique would you recommend?	- Urethra dose-reduction *(dose reduction to urethra below the prescribed dose to the prostate)* - Urethra-steering *(restriction of the hot spots to the urethra without a dose reduction below the prescribed dose to the prostate)* - Both techniques, depending on the clinical situation - I don’t recommend urethra-sparing - Abstain/unqualified to answer	- Both techniques, depending on the clinical situation (56.5 %) - Urethra-steering (30.4 %) - Urethra dose-reduction (13 %)	−
In which clinical situation would you prefer to use a “urethra dose-reduction” modality *(dose reduction to urethra below the prescribed dose to the prostate)*? (multiple choices accepted)	- Conventional fractionation / moderate hypofractionated prostate radiotherapy - Conventional fractionation / moderate hypofractionated prostate radiotherapy with dose-escalation on the DIL - Prostate SBRT - Prostate SBRT with dose-escalation on the DIL - Salvage prostate re-irradiation with SBRT - I don’t recommend this modality - Abstain/unqualified to answer	- Salvage prostate re-irradiation with SBRT (70 %) - Prostate SBRT with dose-escalation on the DIL (48 %) - Prostate SBRT (35 %) - Conventional fractionation / moderate hypofractionated prostate radiotherapy with dose-escalation on the DIL (22 %) - I don’t recommend this modality (13 %) - Conventional fractionation / moderate hypofractionated prostate radiotherapy (0 %) - Abstain/unqualified to answer (0 %)	**Salvage prostate reirradiation with SBRT (70 %)**
In which clinical situation would you prefer a “urethra-steering” modality *(restriction of the hot spots to the urethra without a dose reduction below the prescribed dose to the prostate)*? (multiple choices accepted)	- Conventional fractionation / moderate hypofractionated prostate radiotherapy - Conventional fractionation / moderate hypofractionated prostate radiotherapy with dose-escalation on the DIL - Prostate SBRT - Prostate SBRT with dose-escalation on the DIL - Salvage re-irradiation with SBRT - Postoperative focal SBRT I don’t recommend this modality - Abstain/unqualified to answer	- Prostate SBRT (70 %) Prostate SBRT with dose-escalation on the DIL (70 %) - Salvage re-irradiation with SBRT (56 %) - Conventional fractionation / moderate hypofractionated prostate radiotherapy with dose-escalation on the DIL (39 %) - Conventional fractionation / moderate hypofractionated prostate radiotherapy (13 %) - I don’t recommend this modality (0 %) - Abstain/unqualified to answer (0 %)	**Prostate SBRT (70 %)** **Prostate SBRT with dose-escalation on the DIL (70 %)**
**Urethra definition and additional OARs**			
Which modality do you recommend to define the urethra?	- Foley catheter at the time of simulation CT - MRI - Urethrogram - Surrogate CT-based model (as deep learning-based segmentation) - Other modality - Abstain/unqualified to answer	- MRI (83 %) - Foley catheter at the time of simulation CT (13 %) - Urethrogram (4 %) - Surrogate CT-based model (as deep learning-based segmentation) (0 %) - Other modality (0 %) - Abstain/unqualified to answer (0 %)	**MRI (83 %)**
If MRI: specify sequence and orientation	T2 weighted sequences (78 %) T2 SPIR (fat signal saturation) (11 %) 3D SPACE (*Sampling Perfection with Application optimized Contrast using different flip angle Evolution*) (11 %)		
If the urethra is defined using a Foley catheter, should the treatment also be realized using a Foley catheter?	- Yes - No - Abstain/unqualified to answer	- Yes (48 %) - No (30 %) - Abstain/unqualified to answer (22 %)	−
What urethral portion would you consider sparing?	- Intraprostatic - Bulbous - Membranous - All 3 portions - Only the portions included in the PTV - Abstain/unqualified to answer	- Only the portions included in the PTV (48 %) - All 3 portions (35 %) - Intraprostatic (13 %) - Abstain/unqualified to answer (4 %)	−
Do you recommend a planning-risk volume (PRV) around the urethra? (multiple choices accepted)	- Always - Never - Only for selected cases Abstain/unqualified to answer	- Always (48 %) - Only for selected cases (26 %) - Never (17 %) - Abstain/unqualified to answer (9 %)	−
If you recommend a PRV, which expansion margin would you recommend?	- 1 mm - 2 mm - 3 mm - >3 mm - I don’t recommend the use of a PRV - Abstain/unqualified to answer	- 2 mm (35 %) - 3 mm (26 %) - I don’t recommend the use of a PRV (17 %) - 1 mm (13 %) - Abstain/unqualified to answer (9 %)	−
Would you consider sparing other urinary organs at risk in addition to the bladder and urethra?	- Yes - No - Abstain/unqualified to answer	- No (61 %) - Yes (39 %) - Abstain/unqualified to answer (0 %)	**No (61 %)**
If yes: What other urinary organs at risk would you consider sparing? (multiple choices accepted)	- Bladder trigone - Bladder neck - Striated urinary sphincter Other: please specify Abstain/unqualified to answer	- 9 answers - Bladder trigone (100 %) - Bladder neck (44 %) - Striated urinary sphincter (22 %) - Others: abstention	−
**Urethra dose-constraints**			
Which maximal dose (Dmax − EQD_2Gy_, with an α/β = 3 Gy for late urinary toxicity) would you consider for urethra-sparing independently from the NCCN risk class?	- Dmax < 80 Gy EQD_2Gy_ - Dmax 80–90 Gy EQD_2Gy_ - Dmax 90–100 Gy EQD_2Gy_ Dmax > 100–110 Gy EQD_2Gy_ - I limit the hotspots below 105 %/107 % of the prescription dose, independently from the equivalent dose - I apply different maximal doses based on the NCCN risk class - Abstain/unqualified to answer	- I limit the hotspots below 105 %/107 % of the prescription dose, independently from the equivalent dose (43 %) I apply different maximal doses based on the NCCN risk class (13 %) - Dmax < 80 Gy EQD_2Gy_ (13 %) - Dmax 80–90 Gy EQD_2Gy_ (13 %) - Dmax > 100–110 Gy EQD_2Gy_ (9 %) - Dmax 90–100 Gy EQD_2Gy_ (4 %) - Abstain/unqualified to answer (4 %)	−
Only if you answered to Q17 (*Which maximal dose would you consider for urethra-sparing independently from the NCCN risk class*?) “I apply different maximal doses based on the NCCN risk class”, which maximal dose (Dmax − EQD_2Gy_, with an α/β = 3 Gy for late urinary toxicity) would you consider for urethra-sparing performed for low- to intermediate-risk PCa?	- Dmax < 80 Gy EQD_2Gy_ - Dmax 80–90 Gy EQD_2Gy_ - Dmax 90–100 Gy EQD_2Gy_ Dmax > 100–110 Gy EQD_2Gy_ - I limit the hotspots below 105 %/107 % of the prescription dose, independently from the equivalent dose - Abstain/unqualified to answer	- Abstain/unqualified to answer (50 %) - I limit the hotspots below 105 %/107 % of the prescription dose, independently from the equivalent dose (25 %) Dmax < 80 Gy EQD_2Gy_ (25 %)	−
Only if you answered to Q17 (*Which maximal dose would you consider for urethra-sparing independently from the NCCN risk class*?) “I apply different maximal doses based on the NCCN risk class”, which maximal dose (Dmax − EQD_2Gy_, with an α/β = 3 Gy for late urinary toxicity) would you consider for urethra-sparing performed for high-risk PCa?	- Dmax < 80 Gy EQD_2Gy_ - Dmax 80–90 Gy EQD_2Gy_ - Dmax 90–100 Gy EQD_2Gy_ Dmax > 100–110 Gy EQD_2Gy_ - I limit the hotspots below 105 %/107 % of the prescription dose, independently from the equivalent dose - Abstain/unqualified to answer	- Abstain/unqualified to answer (56 %) - Dmax 80–90 Gy EQD_2Gy_ (25 %) - Dmax 90–100 Gy EQD_2Gy_ (0 %) - Dmax > 100–110 Gy EQD_2Gy_ (0 %) - I limit the hotspots below 105 %/107 % of the prescription dose, independently from the equivalent dose (19 %)	−
In a plan optimization for organs at risk, do you recommend the same plan optimization priority for bladder and urethra?	- Yes, bladder and urethra have the same priority - No, bladder has the priority - No, urethra has the priority - I don’t optimize on urethra - Abstain/unqualified to answer	- Yes, bladder and urethra have the same priority (44 %) - No, bladder has the priority (30 %) - No, urethra has the priority (17 %) - Abstain/unqualified to answer (9 %)	−

**Abbreviations:** Dmax: maximal dose, Gy: Gray, fx: fraction, DIL: dominant intraprostatic lesion, EDQ_2Gy_: equivalent dose for 2 Gy/fx, NCCN: national comprehensive cancer network, PCa: prostate cancer, SBRT: stereotactic body radiotherapy, TURP: transurethral resection of the prostate, CT: computed tomography, PTV: planning target volume, MRI: magnetic resonance imaging.

### Clinical situations

The majority of experts currently use urethra-sparing techniques in prostate cancer treatment, either for all patients (22 %) or only in selected cases (56 %). Experts agreed to consider urethra-sparing depending on radiotherapy technique and fractionation (61 %). While the baseline clinical status (previous transurethral resection of the prostate − TURP, use of α-blockers, etc.) or the National Comprehensive Cancer Network (NCCN) disease risk class do not determine the use of urethra-sparing, tumors located less than 2 mm from the urethra and/or transitional zone and T4 tumors (directly invading the intraprostatic or membranous urethra, external urethral sphincter or bladder) are considered by experts as contraindications for urethra-sparing (70 % and 61 % agreement, respectively). Experts agreed to recommend urethra-sparing whether treating only the prostate gland or including the pelvic nodal regions.

Regarding radiotherapy regimens, urethra-sparing techniques is routinely implemented in the clinical practice for prostate SBRT with focal boosting to the intraprostatic dominant lesion (DIL) (91 % of the experts). Experts agreed to spare urethra for whole gland standard-dose SBRT treatments or for salvage prostate SBRT reirradiation (70 % and 78 % of the experts, respectively). No specific urethra-sparing modality is preferred (urethra-steering or urethra dose-reduction), and both were considered as valuable options. On the other hand, urethra dose-reduction was the preferred technique in patients who were candidates to receive salvage prostate SBRT reirradiation, while there was agreement on the use of urethra-steering techniques for definitive prostate SBRT and prostate SBRT with dose-escalation on the DIL.

### Urethra definition and additional OARs

The majority of experts (83 %) define the urethra using MRI findings, mostly based on T2-weighted sequences. Approximately 11 % of experts use dedicated MRI sequences, such as T2-weighted SPIR or 3D SPACE sequences. Only 13 % of the experts still define the urethra using a Foley catheter at the time of simulation computed tomography (CT). There was no agreement on the use of a Foley catheter during treatment delivery when inserted at time of simulation CT, with 48 % of all experts in favor and 30 % against this approach. For definitive EBRT treatments, most experts (48 %) favored sparing only the urethral portions included in the planning target volume (PTV), while 35 % supported sparing all three urethral portions (intraprostatic, membranous, and bulbous). Seventy-two percent of experts implement a planning organ at risk volume (PRV) around the urethra, with 48 % advocating for its use in all cases and 26 % suggesting it for selected cases. There was considerable variability in the recommended PRV margins around the urethra, with 35 % of experts suggesting a 2-mm expansion, 26 % recommending a 3-mm expansion, and 13 % favoring a 1-mm margin. When asked about sparing other urinary OARs besides the bladder and urethra, 61 % of experts agreed not to spare additional urinary organs. Among the experts who considered other urinary OARs (39 %), all identified the bladder trigone as an organ at risk. Additionally, 44 % proposed the bladder neck, and 22 % proposed the striated urinary sphincter.

### Urethra dose-constraints

**Fig. 2:**
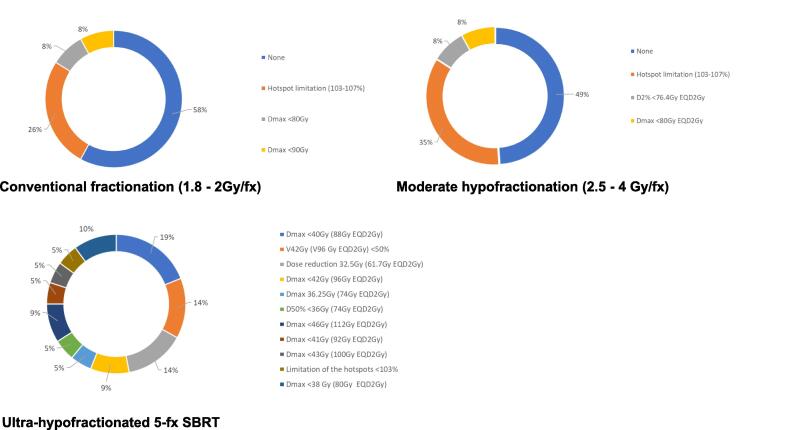
Urethra dose-constraints recommended for prostate cancer radiotherapy using conventional fractionation, moderate hypofractionation, and ultra-hypofractionation (5 fractions SBRT). *Abbreviations:* D_max_ = maximal dose, D_2%_= dose received by 2 % of the urethra, D_50%_= dose received by 50 % of the urethra, EQD_2Gy_ = equivalent dose in 2 Gy per fraction *(EQD_2Gy_ is calculated with a α/β ratio of 3 Gy).*

Large variations existed regarding urethra dose-constraints, highlighting the heterogeneity in practices concerning both EBRT schedules and urethra-sparing strategies. When asked about the maximal dose to consider for urethra-sparing, independently from the NCCN risk class, 43 % of experts preferred limiting hotspots to below 105 % / 107 % of the prescription dose, irrespective of the equivalent dose. For the majority of the panelists, there was no clear indication in using different urethral dose constraints based on the NCCN risk class, with an abstention rate of 50 % and 56 % in treating low-/intermediate-risk and high-risk disease, respectively. For plan optimization purposes, 30 % of experts prioritize bladder dose constraints, while 44 % consider bladder and urethra to have equal priority. A minority, 17 %, prioritize urethra dose constraints over bladder constraints. When considering urethra dose constraints based on fractionation, most experts would not apply specific dose constraints to the urethra for either conventional (58 %) or moderate hypofractionation (49 %) regimens (Fig. 2). Limiting hotspots was the second most favored option for both conventional and moderate hypofractionation, proposed by 26 % of experts for conventional hypofractionation and 35 % for moderate hypofractionation. For a 5-fraction SBRT, no consensus was found on a specific dose-constraint (Fig. 2). Among the experts, 19 % of them proposed a D_max_ (maximal dose received by the urethra) < 40 Gy according to the MIRAGE trial [6], 14 % of them proposed a dose-reduction at 32.5 Gy on the urethra according to the NOVALIS trial [7], [8], and 14.3 % of them proposed a V42Gy (volume of urethra receiving 42 Gy) < 50 % according to the PACE-B trial [9]. Overall, 53 % of the experts implement a D_max_ < 90 Gy EQD_2Gy_ as dose constraint for urethra.

## Discussion

This survey represents the first evaluation of urethra-sparing practices among international experts and marks an effort towards standardizing urethra-sparing practices for prostate cancer EBRT. Despite the relatively small number of international experts involved, which may have affected the robustness of the results, the vast majority of panelists supported the implementation of urethra-sparing for prostate SBRT with focal boosting. Standard dose whole-gland SBRT treatments and prostate salvage SBRT reirradiation represent other clinical situations in which urethral sparing is considered essential. On the other hand, no clear trend was observed regarding urethral dose constraints, highlighting the variability in prostate SBRT schedules and urethra-sparing approaches used in the different centers.

Based on current evidence suggesting a strong correlation between radiation dose to the urethra and urinary toxicity [3], [4], urethra-sparing approaches represent undoubtedly an innovative strategy for mitigating the risk to develop severe urinary toxicity after prostate SBRT [5], [10]. While urethra-sparing is mostly associated with the use of SBRT, specific baseline clinical situations may deserve specific consideration, independently from the fractionation schedule. A history of TURP has been associated with an increased risk of late urinary toxicity, mainly driven by an increased risk of hematuria [5], [11], [12], [13], [14]. In these cases, urethra or even TURP cavity-sparing could be a promising strategy for reducing the risk of late toxicity [15], although specific dose constraints for such approaches are currently lacking. Regarding urethral delineation, T2-weighted MRI sequences represent the preferred delineation modality, although this may be subject to greater interobserver variability than the use of CT-urethrogram [16] or urinary catheter.

Urethra-steering (limitation of hot spots to the urethra) was voted as the preferred modality of urethra sparing for patients receiving definitive prostate SBRT, independently of the use, or not, of focal boosting. Although no formal urethral dose constraints have been validated among experts, the preferred constraints are those used in published prospective trials like MIRAGE [6], PACE-B [9], and NOVALIS [7]. Overall, 53 % of the experts recommend to limit the dose to the urethra to a D_max_ < 90 Gy EQD_2Gy_. While 74 % of experts prioritize dose constraints to the bladder, 44 % believe that dosimetric optimization for both the bladder and urethra should be given equal priority. Most panelists agree that urethra sparing should either target only the urethral portions within the PTV (48 %) or all three urethral portions (35 %), which is especially important for SBRT techniques using non-coplanar beams. A PRV is used by 74 % of experts, though the isotropic margin around the urethra varied between 1 and 3 mm. This variation likely reflects the use of specific image-guided and delivery techniques [1], as well as the growing evidence supporting adaptive MRI-based approaches [8]. Although optimization to urethra represents a promising way to reduce radiation-induced GU toxicities, sparing of other structures like bladder trigone may be considered during treatment planning, as considered by 39 % of the experts. Of note, in the MIRAGE trial, dose delivered to the trigone was associated with an increased risk of GU toxicity after a MR-guided SBRT [17]. Prospective studies are urgently needed to assess the impact of the radiation dose on urinary OARs and its relationship with the development of severe GU toxicity, with the goal of establishing new dose constraints for definitive prostate cancer EBRT.

Experts agreed on the implementation of urethra dose-reduction (dose to the urethra lower than the dose of prescription to the target volume) for salvage prostate reirradiation with SBRT. Nevertheless, published data correlating the dose to the urethra and occurrence of GU toxicity are lacking in the reirradiation setting. To date, few urethra dose-constraints have been proposed for salvage reirradiation. In the phase I GETUG-AFU31 trial, Pasquier *et al*. limited the dose to the urethra PRV (3-mm margin around urethra) to a D_max_ < 39 Gy (V_36Gy_ < 1cc) and V_24Gy_ < 30 % using a 36 Gy in 6 fractions schedule [18], and Bergamin *et al.* used a D_max_ on both urethra and urethra PRV(2-mm margin) at 33 Gy and 36 Gy, respectively [19]. The different toxicity scales (RTOG/EORTC and CTCAE) used, the small numbers of patients, and the short follow-up of these studies preclude any reliable comparison. Fuller *et al.* proposed a 5-fraction schedule delivering 34 Gy in 5 fractions with a high-dose rate (HDR)-like SBRT schedule [20] and limiting the dose to the urethra to a D_max_ < 120 % and D_50%_<105 % of the prescription dose (<40.8 Gy and 35.7 Gy, respectively). The urethra was defined with a Foley catheter. After a follow-up of 44 months Fuller *et al*. reported respectively a 17 % and 8 % rate of late grade ≥ 2 and grade ≥ 3 toxicity [20].

Given the potential for a dose–effect relationship for intraprostatic urethra, even in the reirradiation setting, use of urethral dose reduction strategies, as proposed by Zilli *et al.* in the NOVALIS phase II randomized trial, could probably represent a valid strategy to mitigate toxicity even in the salvage setting [7]. A dose reduction urethra sparing approach delivering 32.5 Gy in 5 fractions to the urethra PRV (2 mm margin) (equivalent to 74 Gy for microscopic disease tumor control) resulted in very low GU toxicity without compromising long-term disease control in patients diagnosed with *de-novo* prostate cancer.

## Conclusions

In modern radiotherapy, urethra sparing is gaining recognition as a promising approach to reduce urinary toxicity in prostate cancer patients undergoing EBRT. According to the current survey, its clinical application is primarily confined to ultra-hypofractionation and reirradiation with SBRT, rather than being guided by patient or disease characteristics. However, the lack of consensus on specific urethra dose constraints and best sparing techniques highlights the need for further research in this field to standardize practices.

## CRediT authorship contribution statement

**Jennifer Le Guévelou:** Conceptualization, Investigation, Methodology, Writing – original draft, Formal analysis. **Paul Sargos:** Conceptualization, Methodology, Writing – review & editing, Investigation. **Piet Ost:** Conceptualization, Methodology, Writing – review & editing, Investigation. **Filippo Alongi:** Writing – review & editing, Investigation. **Stefano Arcangeli:** Writing – review & editing, Investigation. **Alejandro Berlin:** Writing – review & editing, Investigation. **Pierre Blanchard:** Writing – review & editing, Investigation. **Anna Bruynzeel:** Writing – review & editing, Investigation. **Olivier Chapet:** Writing – review & editing, Investigation. **Alan Dal Pra:** Writing – review & editing, Investigation. **Robert T. Dess:** Writing – review & editing, Investigation. **Matthias Guckenberger:** Writing – review & editing, Investigation. **Andrew Loblaw:** Writing – review & editing, Investigation. **Amar U. Kishan:** Writing – review & editing, Investigation. **Barbara Jereczek-Fossa:** Writing – review & editing, Investigation. **David Pasquier:** Writing – review & editing, Investigation. **Mohamed Shelan:** Writing – review & editing, Investigation. **Shankar Siva:** Writing – review & editing, Investigation. **Alison C. Tree:** Writing – review & editing, Investigation. **Costantinos Zamboglou:** Writing – review & editing, Investigation. **Stephane Supiot:** Conceptualization, Methodology, Writing – review & editing, Investigation. **Vedang Murthy:** Conceptualization, Methodology, Writing – review & editing, Investigation. **Thomas Zilli:** Conceptualization, Methodology, Writing – original draft, Formal analysis, Supervision.

## Declaration of competing interest

The authors declare that they have no known competing financial interests or personal relationships that could have appeared to influence the work reported in this paper.
